# Enhancing patient-reported outcomes in stroke care: a path to improved well-being

**DOI:** 10.3389/fneur.2025.1610393

**Published:** 2025-09-12

**Authors:** Irene L. Katzan, Nicolas R. Thompson, Ken Uchino, Brittany Lapin

**Affiliations:** ^1^Center for Outcomes Research and Evaluation, Neurological Institute, Cleveland Clinic, Cleveland, OH, United States; ^2^Cerebrovascular Center, Neurological Institute, Cleveland Clinic, Cleveland, OH, United States; ^3^Department of Quantitative Health Sciences, Cleveland Clinic, Cleveland, OH, United States

**Keywords:** stroke, recovery, patient-reported outcomes, quality of life, intervention – behavioral

## Abstract

Patient-reported outcomes (PROs) present a valuable opportunity to enhance stroke care by capturing symptoms and experiences often missed by traditional outcome measures like the modified Rankin Scale. Despite similar clinician-reported scores, stroke survivors frequently experience varied symptoms across physical, emotional, and social domains that significantly impact their well-being. This commentary examines the evolving role of PROs in stroke care, highlighting their potential to guide personalized treatment strategies. We present cases demonstrating how PROs reveal meaningful clinical differences among seemingly similar patients and discuss implementation challenges in clinical practice. While barriers exist, including time constraints and the complexity of post-stroke symptoms, solutions such as specialized recovery clinics and digital health programs could help bridge the gap between identifying patient needs and delivering targeted interventions. As stroke care evolves, incorporating PROs may unlock new opportunities for improving outcomes by addressing the comprehensive needs of stroke survivors throughout their recovery journey.

## Perspective

Patient-reported outcomes (PROs) are increasingly recognized as vital tools for assessing health status across various conditions, including cardiovascular disease and stroke. While their application in stroke care has received less attention than in cardiovascular medicine, the potential for improving patient care is equally profound. This commentary examines the evolving landscape of PROs in stroke management and their potential to improve care delivery and enhance well-being of stroke survivors.

Stroke patients often experience a range of symptoms that stem from both direct tissue injury and the emotional impact of the event. The modified Rankin Scale (mRS) is a clinician-reported scale widely used for stroke outcome assessment and categorizing the severity of disability but limited in its use as an aide to guide individual medical management (1). As a single-item scale with only seven levels, it provides a restricted framework for evaluating post-stroke experiences. Its heavy emphasis on ambulation overlooks other crucial recovery aspects, failing to capture the diverse symptomatology and varying severities within each level (2). Notably, even patients who achieve a favorable mRS score of 0–2, indicating no to mild disability, commonly report impaired physical function, reduced satisfaction with social roles, cognitive challenges, troublesome fatigue, anxiety and depressive symptoms (3). These “hidden” symptoms often remain undetected in routine clinical assessments unless specifically explored, making PROs an invaluable tool for comprehensive symptom evaluation.

### PROs in stroke clinical research

PROs have occasionally been used as secondary endpoints in stroke trials, most commonly the EuroQol 5 Dimension (EQ-5D) utility index. Recently, the EQ-5D utility index has been integrated with the mRS to create the utility-weighted mRS (UW-mRS), which converts mRS scores into the mean EQ-5D utility values for stroke patients at each level (4). This approach provides a rough estimate of health-related quality of life (hrQoL) while addressing the ordinal limitations of the mRS commonly encountered in clinical trials. Health utility weights from other scales have occasionally been used to determine the UW-mRS. Additionally, recent stroke trials have begun to include Patient Reported Outcome Measurement Information System (PROMIS) and Quality of Life in Neurological Disorders (Neuro-QoL) scales as secondary outcomes to assess hrQoL (5–7).

### PROs in clinical care

While the UW-mRS represents an advancement, its lack of granularity, along with that of the EQ-5D restricts their ability to pinpoint specific issues that require attention for individual patients. This limitation makes them ineffective tools for guiding personalized management strategies in stroke care.

Because of the broad and heterogenous symptoms experienced by stroke patients, which encompass physical, emotional, cognitive, and social health domains, the PRO measures that have been typically used to assess health status incorporate multiple domains, such as the Short Form-36 (SF-36), and Stroke Impact Scale. The Patient Reported Outcome Measure Information System Global Health (PROMIS GH) scale has been recommended by the International Consortium for Health Outcomes Measurement (ICHOM) for assessing outcomes in stroke patients (8). It consists of 10 items, each assessing a different domain of health, which can be used individually or combined into physical or mental health summary scores. In addition to PROMIS GH, other PROMIS tools and the related Neuro-QoL tools offer separate domain-specific scales that provide a more precise evaluation of health domains (9) and have been effectively used in the assessment of stroke patients (3, 10, 11). One significant advantage of these tools is their availability in computer-adaptive testing (CAT) formats, which allows for a more efficient and targeted assessment of specific health domains while minimizing ceiling and floor effects. T-scores are standardized to the adult U. S. population, providing an easily interpretable score across domains. The Neuro-QoL tools were developed using data from stroke patients, and the psychometric properties of PROMIS tools have been evaluated in this population, including the determination of thresholds for meaningful change (12–15). These features make these tools well-suited for capturing the comprehensive health profiles of stroke survivors.

The Table 1 demonstrates the utility of PROs in identifying clinically significant symptom differences among outpatient ischemic stroke survivors seen at Cleveland Clinic. It shows PRO data on three male patients, aged 60–65 years, who had experienced ischemic strokes within the previous 2 months. As part of routine care, they completed several PROMIS CAT scales and the Patient Health Questionnaire-9 (PHQ-9) depression scale. PROMIS scores are displayed to the providers as percentiles to simplify interpretation. Despite similar mRS and National Institutes of Health Stroke Scale (NIHSS) scores, both indicating good neurological recovery, their PRO results revealed marked differences in post-stroke experiences.

**Table 1:** Heterogeneity of patient-reported outcomes of three male patients of similar age with recent stroke and minimal to no deficits on clinician-reported measures.

Clinical information	Patient A	Patient B	Patient C
Symptoms at initial presentation	Balance difficulty	Visuospatial difficulties	Right visual field deficit
Stroke mechanism	Small-vessel disease	Intracranial stenosis	Intracranial stenosis
Comorbid conditions	Hypertension, smoker	Hypertension, diabetes	Heart failure, hypertension, hyperlipidemia
Clinician-reported scales^†^			
NIHSS	0	0	1
Modified Rankin scale	1	0	0
PROMIS/Neuro-Qol Percentiles^‡^			
Physical health			
Physical function	3**	77	38
Fatigue	3**	99	19*
Sleep disturbance	1**	82	10**
Pain interference	3**	87	29*
Social health			
Satisfaction social roles	3**	96	55
mental health			
Depression^§^	9**	90	28*
Neuro-QoL cognition	55	96	75
Anxiety	58	70	73
Global health			
Physical health summary	4**	88	10**
Mental health summary	25*	62	33
Provider’s interpretation of questionnaire results	Patient has significant symptoms in multiple domains spanning physical, social, and mental health, which are worse than 95% of population in several domains	No troublesome symptoms identified across measured constructs Patient scores are similar or better than general population in all measured constructs	Patient has symptoms in several domains measuring aspects of physical health His most prominent symptoms relate to sleep disturbance and fatigue; sleep disturbance may be contributing to his fatigue He is experiencing pain, as well, which may be exacerbated by or contribute to poor sleep.
Potential management implications	May benefit from referral to behavioral health Consider exploring interventions for sleep and pain symptoms Consider additional physical therapy	Provider can focus on secondary risk factor management	Consider exploring sleep issues Inquire about the impact of pain on daily functioning and sleep

Score interpretation that is provided as Table footnote to providers: Percentiles provide an indication of how a patient’s score ranks in relation to the U. S. general population. ≥ 31st percentile is within normal limits or better. * < 31st percentile is ≥ ½ SD worse than population, may be clinically relevant. ** < 16th percentile is ≥ 1 SD worse than population, warrants attention. † Clinician-reported scales completed at the office visit; ‡ All are PROMIS scales except NeuroQoL Cognitive Function. Score percentiles areÝisplayed instead of T-scores to simplify clinical interpretation; § Depressive symptoms were assessed with Patient Health Questionnaire - 9 depression scale and are calibrated to the the PROMIS Depression metric.

#### Patient A: comprehensive symptom profile

Patient A had minimal clinical deficits (NIHSS 0, mRS 1), but his PRO scores reveal significant impairment across multiple domains. Scoring below the tenth percentile in seven domains, his experience represents a profound disconnect between clinical assessment and lived reality. This case demonstrates how traditional measures can miss substantial suffering in seemingly well-recovered patients.

#### Patient B: optimal recovery profile

Patient B had PRO scores that align perfectly with his excellent clinical outcomes (NIHSS 0, mRS 0), with most domains scoring above the 80th percentile. His exceptional scores across physical, social, and emotional domains represent an ideal recovery trajectory. This case illustrates how some stroke survivors can achieve comprehensive recovery that is accurately reflected in both clinical measures and PRO assessments.

#### Patient C: domain-specific impairments

Patient C had favorable clinical scores (NIHSS 1, mRS 0), but shows significant impairments in specific domains – most notably sleep disturbance (10th percentile), but also fatigue and pain. This indicates a symptom cluster. This case highlights how stroke can selectively impact health domains and how targeted interventions for sleep might address his constellation of symptoms.

These cases demonstrate how PROs reveal important clinical differences invisible to traditional outcome measures. Patient A requires comprehensive multidisciplinary care despite minimal visible deficits, including potential referrals to behavioral health specialists, detailed evaluation of sleep and pain symptoms, and consideration of additional physical therapy. Patient B needs only standard secondary prevention, allowing efficient resource allocation. Patient C would benefit from targeted sleep interventions such as assessment for sleep apnea or referral to a sleep specialist that might improve his related fatigue and pain symptoms. This heterogeneity in patient experiences underscores the value of PRO data in creating personalized recovery pathways.

#### Barriers and potential solutions to the implementation of PROs in clinical care

Technological advances in electronic health records (EHRs) and patient portals have facilitated the integration of PRO collection into routine clinical care. Modern EHR systems now provide multiple collection modalities that accommodate different clinical practices and varying workflows. Patients can complete questionnaires remotely via smartphones or computers through patient portals, or in-person using electronic tablets and examination room workstations (16, 17). This flexibility ensures that patients without personal devices or internet access can still participate in PRO collection. However, other patient accessibility challenges must be considered. Patients with limited health literacy may struggle with electronic questionnaires, making it necessary for clinical programs to evaluate the reading level and complexity of selected instruments (18). Language barriers present another significant obstacle (19), requiring practices to ensure questionnaire availability in the primary languages of their patient population—a consideration that may limit questionnaire selection options.

Despite the increasing availability of electronic PRO collection within EHRs and the potential benefit of PROs in clinical care, their integration into routine practice faces several significant challenges. Healthcare providers often lack specialized training in interpreting and addressing PRO data in the context of post-stroke care. The complexity of post-stroke symptoms presents a particular challenge, as these symptoms are frequently multifactorial, requiring clinicians to “peel back layers” to identify appropriate management strategies. Interventions that require active patient engagement and behavioral modifications have proven especially difficult to implement successfully, as demonstrated by several negative trials of stroke risk factor control (20–22). Artificial intelligence has the potential to reduce some of the barriers to integration and use of PROs within clinical practice in the future (23–25).

The effective implementation of PROs is further complicated by significant systemic barriers. Time constraints pose a substantial challenge, as clinicians must balance limited appointment durations with competing clinical priorities and documentation requirements. Nevertheless, few objectives surpass the importance of enhancing a stroke survivor’s overall sense of well-being. If the stroke care community could make even modest advances in this arena, it could revolutionize our approach to patient management.

One potential strategy is the implementation of recovery clinics or digital health programs specifically tailored for stroke survivors. These programs could provide structured support systems, standardized assessment protocols, and coordinated care pathways to better address the complex needs identified through PROs. Such specialized programs could help bridge the gap between identifying patient needs through PROs and delivering appropriate, targeted interventions.

The concept of a stroke recovery clinic parallels that of cancer survivorship clinics in several ways, addressing the multifaceted challenges faced by survivors of life-altering medical events. Both cancer and stroke survivors must cope with residual impairments from their medical condition, navigate the emotional impact of their diagnosis, and manage anxiety about potential recurrence. Both models emphasize that recovery extends beyond the acute medical event, necessitating comprehensive support for physical, emotional, and lifestyle adjustments essential for optimal recovery and quality of life. The American College of Surgeons Commission on Cancer mandates that cancer programs include survivorship programs for adults with a high likelihood of being cured, a requirement established in 2021 (26).

Over the past two decades, the concept of “systems of care” in acute stroke treatment has evolved from a nascent idea to the gold standard, with hundreds of hospitals now certified to deliver comprehensive protocols including time-critical intravenous thrombolysis and routine depression screening (27). As we advance stroke care, we posit that the next frontier could lie in post-acute care including the systematic assessment and management of post-stroke symptoms across emotional, social, and physical domains. This evolution is already underway, as evidenced by recent guidelines from the American Heart Association/American Stroke Association Nursing Council emphasizing the critical role of nurses in addressing psychosocial health after stroke (28), alongside the 2022 guidelines statement on comprehensive symptom assessment in stroke survivors (29).

Substantial additional research is required to establish whether systematic PRO collection meaningfully improves health-related quality of life for stroke patients. Key research priorities include identifying the most relevant PROs to measure and determining optimal assessment timepoints. Furthermore, studies must evaluate which interventions should be triggered by specific PRO responses, considering that multiple interventions may be needed simultaneously. Research should also examine whether treatment approaches should be tailored based on stroke severity, residual functional deficits, patient demographics, and comorbid conditions. Understanding these nuances will be critical for developing evidence-based frameworks that translate PRO data into actionable clinical improvements.

If PRO collection is shown to be effective, the future adoption of these programs could be significantly improved by offering reimbursement at the hospital-system level for PRO collection and implementation of appropriate care plans. Billing insurance directly at the patient level has the disadvantage of shifting costs to patients through copays, deductibles, or even direct charges for those who are uninsured or whose insurance does not cover these services. This approach may discourage patients from completing PROs (I. Katzan, personal communication), potentially undermining the program’s effectiveness.

As we advance in post-stroke patient care, incorporating PROs allows us to consider the complex, multifaceted aspects of patient well-being, potentially unlocking new opportunities for significant improvements in outcomes. By broadening our focus beyond physical rehabilitation and risk factor management, we can develop interventions that address the comprehensive needs of stroke survivors throughout their recovery journey.

## Data Availability

The raw data supporting the conclusions of this article will be made available by the authors, without undue reservation.
